# Adverse event prediction in propofol-remimazolam tosilate anesthesia

**DOI:** 10.3389/fmed.2025.1731100

**Published:** 2026-01-15

**Authors:** Minmin Zhai, Fengqiang Sun, Shengyong Liang

**Affiliations:** Department of Anesthesiology, Feicheng People’s Hospital, Taian, China

**Keywords:** anesthesia-related adverse events, machine learning, propofol, random forest model, remimazolam tosilate

## Abstract

**Objective:**

This study aimed to develop and validate a predictive model for anesthesia-related adverse events (ARAEs) in patients receiving propofol combined with remimazolam tosilate, based on perioperative clinical indicators.

**Methods:**

A retrospective study was conducted on patients who underwent propofol-remimazolam tosilate anesthesia at our hospital from January 2021 to December 2024. The cohort was divided into a training set (*n* = 218, 70%) and a validation set (*n* = 94, 30%). Demographic characteristics, vital sign monitoring data, laboratory test results, and anesthesia recovery parameters were collected. Independent predictors of ARAEs were identified through univariate and multivariate logistic regression analyses. Machine learning algorithms, including random forest (RF), support vector machine, and gradient boosting, were employed to construct predictive models. Model performance was assessed using the area under the receiver operating characteristic curve (AUC), calibration curves, and decision curve analysis (DCA). The optimal model was selected, and feature importance was analyzed.

**Results:**

No significant differences were observed in baseline characteristics between the training and validation sets (*P* > 0.05). Univariate analysis and multivariate logistic regression identified surgical duration, intraoperative hypotension incidence, spontaneous breathing recovery time, serum creatinine, and arterial carbon dioxide partial pressure as independent risk factors for ARAEs (all *P* < 0.05). Among the machine learning models, the RF model demonstrated the highest discriminative ability in both the training (AUC 0.814, 95% CI: 0.738–0.889) and validation sets (AUC 0.777, 95% CI: 0.640–0.913), along with superior calibration and clinical net benefit. Feature importance analysis showed that surgical duration, and anesthetic drug dosage ratio were the most critical predictive factors.

**Conclusion:**

The RF model, developed using key perioperative indicators, effectively predicts the risk of ARAEs during propofol-remimazolam tosilate anesthesia. Surgical duration, hemodynamic stability, and respiratory recovery status are the most significant predictors.

## Introduction

General anesthesia is essential for the successful implementation of surgical procedures, and the rational use of anesthetic agents is closely associated with perioperative patient safety (1). Propofol, a short-acting intravenous anesthetic, offers rapid onset and high-quality recovery, while remimazolam tosilate, a novel benzodiazepine derivative, exhibits metabolism independent of hepatic function. The combined use of these two agents can optimize the anesthesia process to some extent (2). However, in clinical practice, some patients may still experience anesthesia-related adverse events (ARAEs), including hemodynamic instability, prolonged respiratory depression, and delayed recovery, which not only affect operating room efficiency but also increase the risk of postoperative complications, posing a significant challenge in anesthesia management (3).

Currently, the assessment of ARAEs primarily relies on real-time intraoperative observation and empirical judgment by anesthesiologists, lacking objective and quantifiable early warning tools for effective preemptive intervention (4). Recent studies suggest that ARAEs result from complex interactions among patient baseline status, surgical stress intensity, and pharmacological effects of anesthetics (5). For example, surgical duration directly reflects trauma and stress duration, intraoperative hypotension incidence indicates circulatory instability, and spontaneous breathing recovery time reflects respiratory depression severity, while serum creatinine and arterial carbon dioxide partial pressure (PaCO_2_) indirectly indicate renal function and ventilation efficiency, respectively (6). However, individual clinical parameters have limited predictive value, and traditional statistical methods struggle to capture their non-linear interactions.

Machine learning techniques, capable of efficiently processing multidimensional and non-linear clinical data, demonstrate strong potential in risk prediction and pattern recognition, gradually gaining traction in perioperative risk assessment. Therefore, this study aims to integrate key perioperative vital signs, laboratory tests, and anesthesia recovery indicators to construct predictive models for ARAEs associated with propofol-remimazolam tosilate anesthesia using multiple ML algorithms (random forest, support vector machine, and gradient boosting), providing an objective and precise risk assessment tool to facilitate personalized and proactive anesthesia management.

## Materials and methods

### Study population

This was a retrospective observational study conducted on patients who underwent propofol-remimazolam tosilate anesthesia at our hospital from January 2021 to December 2024. All of them gave informed consent and voluntarily participated in this study, and the study was approved by the hospital’s ethics committee. The patients were randomly divided into a training set and a validation set at a ratio of 7:3.

Inclusion criteria: (1) Elective general anesthesia with propofol-remimazolam tosilate; (2) American Society of Anesthesiologists (ASA) physical status I–III; (3) Age 18–75 years; (4) Complete anesthesia records and perioperative monitoring data; (5) Preoperative laboratory results available.

Exclusion criteria: (1) Severe hepatic/renal dysfunction (Child-Pugh B/C or eGFR < 30 mL/min/1.73 m^2^). (2) Difficult airway, severe respiratory disease, or obstructive sleep apnea. (3) Chronic use of sedatives/analgesics/psychotropic drugs. (4) Emergency/post-CPR surgery. (5) Pregnancy or lactation. (6) Incomplete clinical data.

### Sample size calculation

Sample size calculation was performed based on the primary outcome (ARAEs under propofol-remimazolam tosilate anesthesia). The expected incidence rate (12–15%) was determined from prior literature on combined propofol-remimazolam anesthesia, preliminary clinical trial data, and institutional surgical population characteristics. Using SPSS 26.0 (logistic regression sample size estimation module) and R 4.2.1 (via the pwr and powerMediation packages), we ensured statistical power adequacy with the following parameters: significance level (α = 0.05, two-tailed), power (1-β = 80%), and a 10% dropout rate. The minimum required sample size was 312; thus, 345 patients undergoing propofol-remimazolam tosilate anesthesia from January 2021 to December 2024 were enrolled. *Post hoc* analysis confirmed that the actual sample size met the statistical requirements (power > 80%, EPV ≥ 10:1 in multivariate logistic regression).

A total of 345 patients were initially enrolled. After applying exclusion criteria and removing cases with incomplete data, 312 patients were included in the final analysis (training set: *n* = 218; validation set: *n* = 94).

### Anesthetic protocol

All patients received intravenous induction of general anesthesia with propofol (1.5–2.0 mg/kg) and remimazolam tosilate (0.3–0.6 mg/kg) administered sequentially. Anesthesia maintenance was achieved with continuous infusion of propofol (4–12 mg⋅kg^–1^⋅h^–1^) and remimazolam tosilate (0.1–0.3 mg⋅kg^–1^⋅h^–1^), adjusted to maintain a mean bispectral index (BIS) value between 40 and 60. Fentanyl (2–4 μg/kg) was used for analgesia, and rocuronium (0.6–0.8 mg/kg) was administered for muscle relaxation when necessary. Intraoperative ventilation was controlled with a tidal volume of 6–8 mL/kg, respiratory rate of 12–16 breaths/min, and end-tidal carbon dioxide partial pressure maintained at 35–45 mmHg. Hemodynamic management: if MAP decreased by more than 20% of baseline, phenylephrine (50–100 μg) was administered intravenously; if heart rate was less than 50 beats/min, atropine (0.5 mg) was given as needed. Compared with single-agent anesthesia, this combined regimen reduces the dosage of each drug, thereby minimizing the cardiovascular and respiratory inhibitory effects of individual anesthetics.

### Data collection

Demographics, intraoperative monitoring, anesthesia recovery, and laboratory data were retrieved from electronic medical records, anesthesia clinical information systems, and laboratory databases.

Variables collected:

Baseline/demographics: Age, gender, body mass index (BMI), ASA classification (7).

Intraoperative monitoring: Surgery duration, BIS, intraoperative hypotension incidence, heart rate (HR) variability, tidal volume (VT) variation, anesthetic drug dosage ratio (propofol/remimazolam).

Recovery indices: orientation recovery time.

Laboratory tests (collected within 24 h before surgery on the day of operation): alanine transaminase (ALT), aspartate transaminase (AST), serum creatinine (Scr), estimated glomerular filtration rate (eGFR), potassium (K^+^), sodium (Na^+^), calcium (Ca^2+^), prothrombin time (PT), activated partial thromboplastin time (APTT), arterial pH, PaCO_2_, arterial oxygen partial pressure (PaO2).

Intraoperative hypotension incidence was defined as the proportion of patients with MAP < 80 mmHg or a 20% reduction from baseline for > 5 min during anesthesia induction or maintenance (8).

### Outcome definition

Per Miller’s Anesthesia (9) and Chinese Society of Anesthesiology guidelines, patients were stratified into:

Adverse event group (meeting ≥ 1 criterion): (1) Severe hemodynamic instability (MAP fluctuation > 30% from baseline for > 10 min or vasopressor use > 2 doses); (2) Respiratory depression (SpO2 < 90% for > 1 min or spontaneous breathing recovery > 10 min); (3) Delayed recovery (orientation recovery > 15 min post-surgery). Events were independently verified by two senior anesthesiologists.

No adverse event group: Patients without any above events.

### Statistical analysis

Data analysis was performed using SPSS 26.0, R 4.2.3, and Python 3.8.5. Normally distributed continuous variables are presented as mean ± standard deviation (x̄ ± SD) and were compared between the training and validation sets or between the adverse event and non-adverse event groups using independent-sample *t*-tests. Non-normally distributed continuous variables are expressed as median (interquartile range) [M (Q1, Q3)] and compared using the Mann-Whitney U test. Categorical variables are presented as numbers (percentages) [n (%)] and analyzed using the χ^2^ test or Fisher’s exact test, as appropriate. Univariate analysis was conducted in the training set to identify potential influencing factors. To optimize the model and prevent overfitting, least absolute shrinkage and selection operator (LASSO) regression was initially applied for variable selection. The selected features were then incorporated into multivariate logistic regression to identify independent risk factors for intraoperative adverse events, with results expressed as odds ratios (ORs) and 95% confidence intervals (95% CI). Based on the independent predictors, random forest (RF), support vector machine (SVM), and gradient boosting (GB) models were employed to construct predictive models. The receiver operating characteristic (ROC) curve was plotted, and the area under the curve (AUC) value was calculated. Calibration was assessed by plotting calibration curves (using the bootstrap method with 1,000 resamples) and performing the Hosmer-Lemeshow goodness-of-fit test. Decision curve analysis (DCA) was used to evaluate the clinical application value of the nomogram by calculating the net benefit at different threshold probabilities. The nomogram prediction model was constructed using the “rms” package in R software. In addition, the SHapley Additive exPlanations (SHAP) values were calculated using the “shap” library in Python to evaluate the model interpretability from both global (feature importance ranking, contribution direction) and local (decomposition of risk contribution for single-case patients) dimensions. Sample size estimation adhered to the “events per variable (EPV)” principle for predictive modeling studies, requiring at least 10 outcome events per candidate predictor. This study ultimately included 5 predictive variables, with 62 adverse events (outcome events) in the training set, meeting the EPV > 10 criterion. Furthermore, low multicollinearity among variables (VIF < 2) confirmed model stability. The significance level was set at α = 0.05.

Continuous variables were standardized using z-score normalization before being input into machine learning models. Missing data (< 2%) were imputed using k-nearest neighbors (*k* = 5).

## Results

### Baseline characteristics of training and validation sets

The training set (*n* = 218) included 62 ARAE cases (28.44%) and 156 controls (71.56%); the validation set (*n* = 94) had 28 ARAE cases (29.79%) and 66 controls (70.21%). No significant differences were observed between sets (*P* > 0.05). Among the 62 adverse events in the training set, the distribution was as follows: hemodynamic instability (*n* = 32, 51.6%), respiratory depression (*n* = 18, 29.0%), and delayed recovery (*n* = 12, 19.4%). A similar distribution was observed in the validation set. The distribution of surgical types was balanced between the training and validation sets, with abdominal surgery being the most common (Table 1).

**TABLE 1:** Baseline characteristics of training and validation sets.

Indicators		Training set (*n* = 218)	Validation set (*n* = 94)	t/χ ^2^	*P*
Age (years)		58.62 ± 9.35	57.98 ± 8.76	0.565	0.572
Gender	Male	125(57.34%)	54(57.45%)	0.005	0.986
	Female	93(42.66%)	40(42.55%)		
BMI (kg/m^2^)		24.35 ± 3.12	23.98 ± 2.95	0.976	0.329
Type of surgery	Abdominal	89(40.8%)	38(40.4%)	0.217	0.975
	Urological	42(19.3%)	18(19.1%)		
	Orthopedic	51(23.4%)	21(22.3%)		
	Gynecological	36(16.5%)	17(18.1%)		
Operation duration (min)		128.56 ± 35.72	125.34 ± 32.18	0.751	0.452
Mean BIS value during anesthesia maintenance		48.35 ± 6.21	47.89 ± 5.98	0.607	0.544
Intraoperative hypotension incidence [n(%)]		68(31.2%)	30(31.9%)	0.016	0.900
HR coefficient of variation (%)		8.35 ± 2.17	8.02 ± 1.98	1.265	0.207
Tidal volume (VT) variation rate (%)		7.85 ± 2.63	7.52 ± 2.31	1.054	0.292
Time for recovery of orientation (min)		10.56 ± 3.25	10.12 ± 2.98	1.479	0.140
Anesthetic drug dosage ratio (propofol/remimazolam)		1.85 ± 0.42	1.79 ± 0.38	1.036	0.301
ALT (U/L)		32.65 ± 10.32	31.89 ± 9.76	0.607	0.545
AST (U/L)		29.45 ± 8.67	28.98 ± 8.12	0.447	0.654
Scr (μmol/L)		78.35 ± 15.21	76.98 ± 14.56	0.739	0.460
eGFR (mL/min⋅1.73 m^2^)		92.56 ± 18.32	90.89 ± 17.65	0.749	0.456
K^+^ (mmol/L)		3.85 ± 0.32	3.79 ± 0.29	1.562	0.119
Na^+^ (mmol/L)		138.65 ± 3.12	137.98 ± 2.89	1.788	0.076
Ca^2+^ (mmol/L)		2.25 ± 0.17	2.21 ± 0.15	1.974	0.066
PT (seconds)		11.85 ± 1.23	11.69 ± 1.18	1.067	0.286
APTT (seconds)		32.65 ± 4.12	31.98 ± 3.87	1.342	0.181
pH		7.38 ± 0.05	7.37 ± 0.04	1.716	0.087
PaCO_2_ (mmHg)		38.65 ± 4.32	37.98 ± 3.98	1.286	0.199
PaO2 (mmHg)		105.35 ± 12.67	103.89 ± 11.98	0.949	0.343

### Univariate analysis of influencing factors for adverse events during propofol combined with remimazolam tosilate anesthesia

In the training set, univariate analysis showed that there were statistically significant differences in surgical duration, intraoperative hypotension incidence, anesthetic drug dosage ratio, serum creatinine, and arterial blood PaCO_2_ between patients in the adverse event group and those in the non-adverse event group (all *P* < 0.05) (Table 2).

**TABLE 2:** Univariate analysis of influencing factors for the occurrence of adverse events during propofol combined with remimazolam tosilate anesthesia.

Indicators		Adverse event group (*n* = 62)	No adverse event group (n = 156)	t/χ ^2^	*P*
Age (years)		58.82 ± 9.39	58.23 ± 9.20	0.424	0.617
Gender	Male	40(64.52%)	85(54.49%)	1.824	0.176
	Female	22(35.48%)	71(45.51%)		
BMI (kg/m^2^)		24.05 ± 3.10	24.39 ± 3.17	0.719	0.473
Type of surgery	Abdominal	28 (45.2%)	61 (39.1%)	1.356	0.716
	Urological	10 (16.1%)	32 (20.5%)		
	Orthopedic	15 (24.2%)	36 (23.1%)		
	Gynecological	9 (14.5%)	27 (17.3%)		
Operation duration (min)		145.56 ± 35.72	120.56 ± 33.01	4.927	0.001
Mean BIS value during anesthesia maintenance		48.25 ± 6.21	48.15 ± 6.12	0.108	0.913
Intraoperative hypotension incidence [n(%)]		32(51.6%)	36(23.1%)	10.745	0.001
HR coefficient of variation (%)		8.38 ± 2.17	8.31 ± 2.11	0.219	0.826
Tidal volume (VT) variation rate (%)		7.89 ± 2.63	7.83 ± 2.60	0.153	0.878
Time for recovery of orientation (min)		10.58 ± 3.27	10.52 ± 3.23	0.123	0.902
Anesthetic drug dosage ratio (propofol/remimazolam)		2.14 ± 0.45	1.68 ± 0.36	7.906	0.001
ALT (U/L)		32.69 ± 10.32	32.60 ± 10.25	0.058	0.954
AST (U/L)		29.50 ± 8.69	29.44 ± 8.61	0.046	0.963
Scr (μmol/L)		85.95 ± 15.21	75.05 ± 14.12	5.029	0.001
eGFR (mL/min⋅1.73 m^2^)		92.58 ± 18.34	92.41 ± 18.25	0.062	0.951
K^+^ (mmol/L)		3.89 ± 0.33	3.82 ± 0.30	1.510	0.132
Na^+^ (mmol/L)		138.75 ± 3.02	138.55 ± 3.01	0.442	0.658
Ca^2+^ (mmol/L)		2.26 ± 0.18	2.24 ± 0.16	0.803	0.423
PT (seconds)		11.88 ± 1.28	11.80 ± 1.20	0.436	0.664
APTT (seconds)		32.68 ± 4.12	32.62 ± 4.11	0.097	0.922
pH		7.38 ± 0.05	7.37 ± 0.04	1.546	0.123
PaCO_2_ (mmHg)		41.95 ± 4.32	37.62 ± 4.32	6.676	0.001
PaO2(mmHg)		105.45 ± 12.68	105.20 ± 12.61	0.132	0.895

### Multivariate logistic regression analysis of influencing factors

**FIGURE 1:**
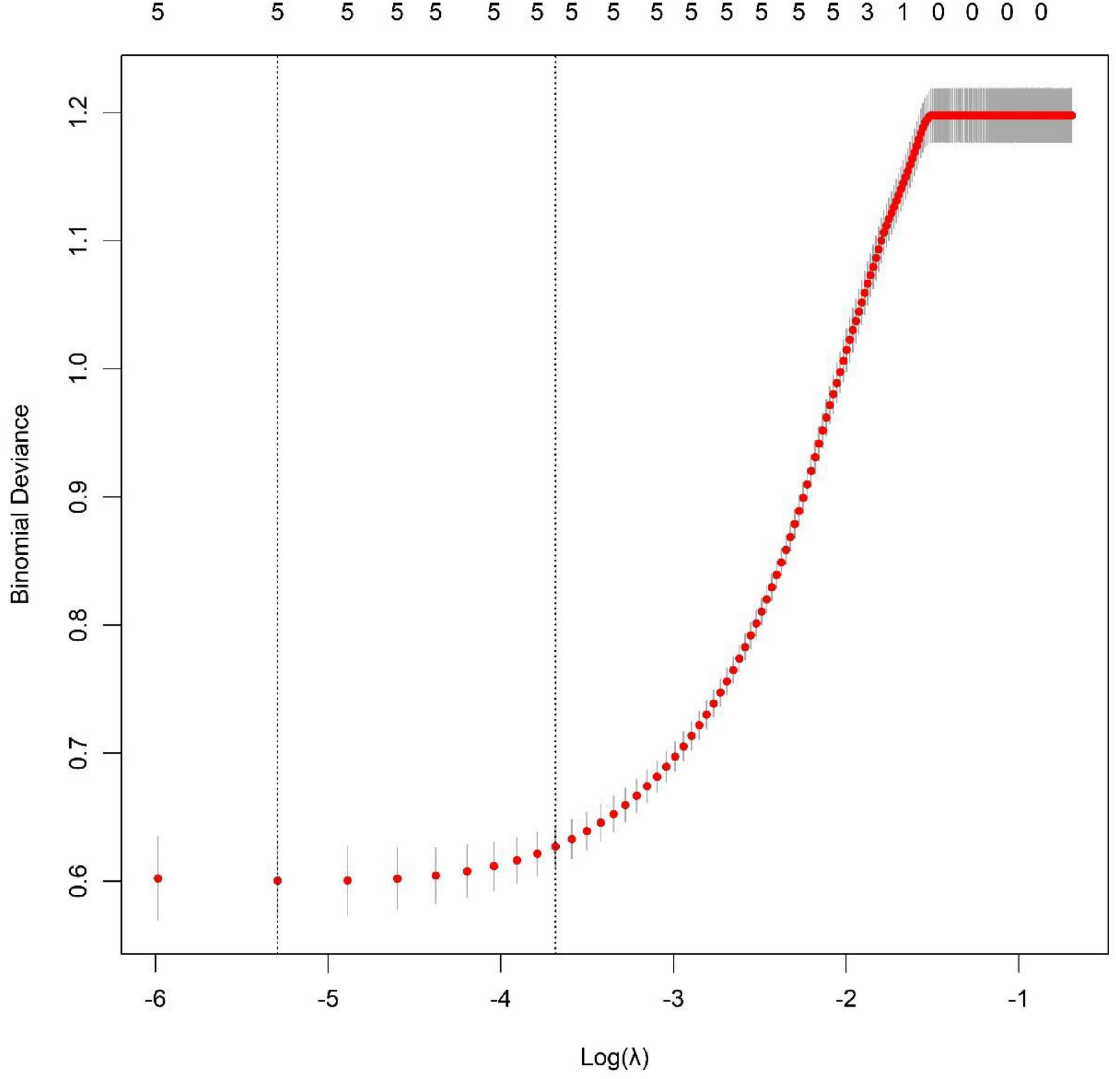
Results of Lasso regression cross—validation.

Whether adverse events occurred during propofol combined with remimazolam tosilate anesthesia in patients was used as the dependent variable (non-adverse event group = 0, adverse event group = 1) (Supplementary Table 1). Five indicators with statistically significant differences in the univariate analysis were included were included in the LASSO regression for variable screening. The optimal variables were selected using 10-fold cross-validation and the λ-1se criterion (Figure 1). Then the screened indicators were included in the multivariate Logistic regression model. The results showed that surgical duration, intraoperative hypotension incidence, Anesthetic drug dosage ratio, Scr, and PaCO_2_ were independent risk factors for the occurrence of adverse events (all *P* < 0.05) (Table 3).

**TABLE 3:** Multivariate Logistic regression analysis of influencing factors for adverse events during propofol combined with remimazolam tosilate anesthesia.

Factor	β	SE	Wald	*P*	OR	95%CI
Surgical duration	0.022	0.007	10.983	0.001	1.022	1.009–1.036
Intraoperative hypotension incidence	0.271	0.059	20.427	0.001	1.314	1.164–1.477
Anesthetic drug dosage ratio (propofol/remimazolam)	1.148	0.029	9.481	0.007	1.564	1.183–1.948
Scr	0.039	0.015	6.570	0.010	1.044	1.009–1.072
PaCO_2_	0.227	0.063	13.065	0.001	1.237	1.113–1.375
Constant	−21.403	3.136	46.580	0.001	0.001	

### Performance evaluation of machine learning models

**FIGURE 2:**
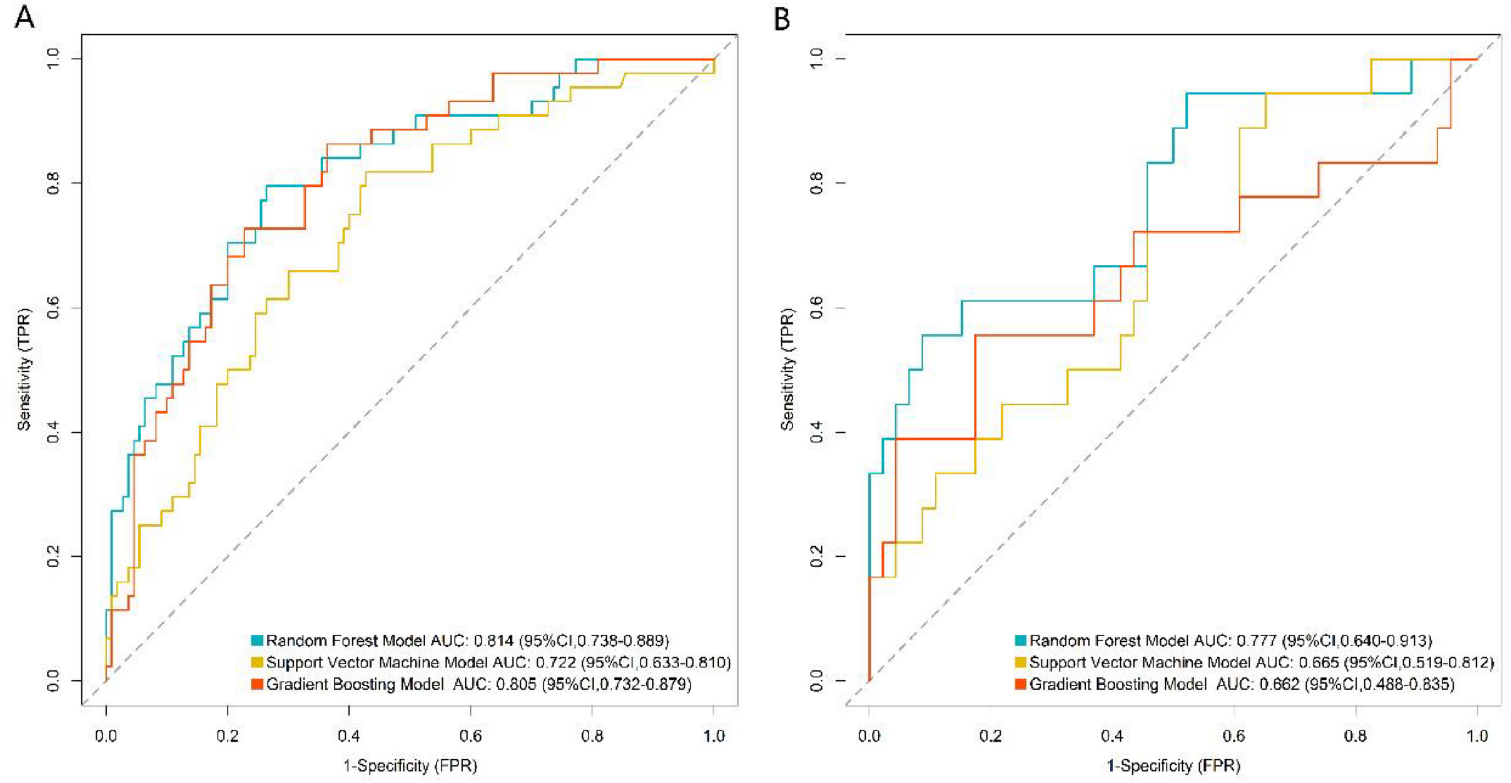
Receiver operating characteristic curves of machine learning models (**A**, the training set; **B**, the validation set).

**FIGURE 3:**
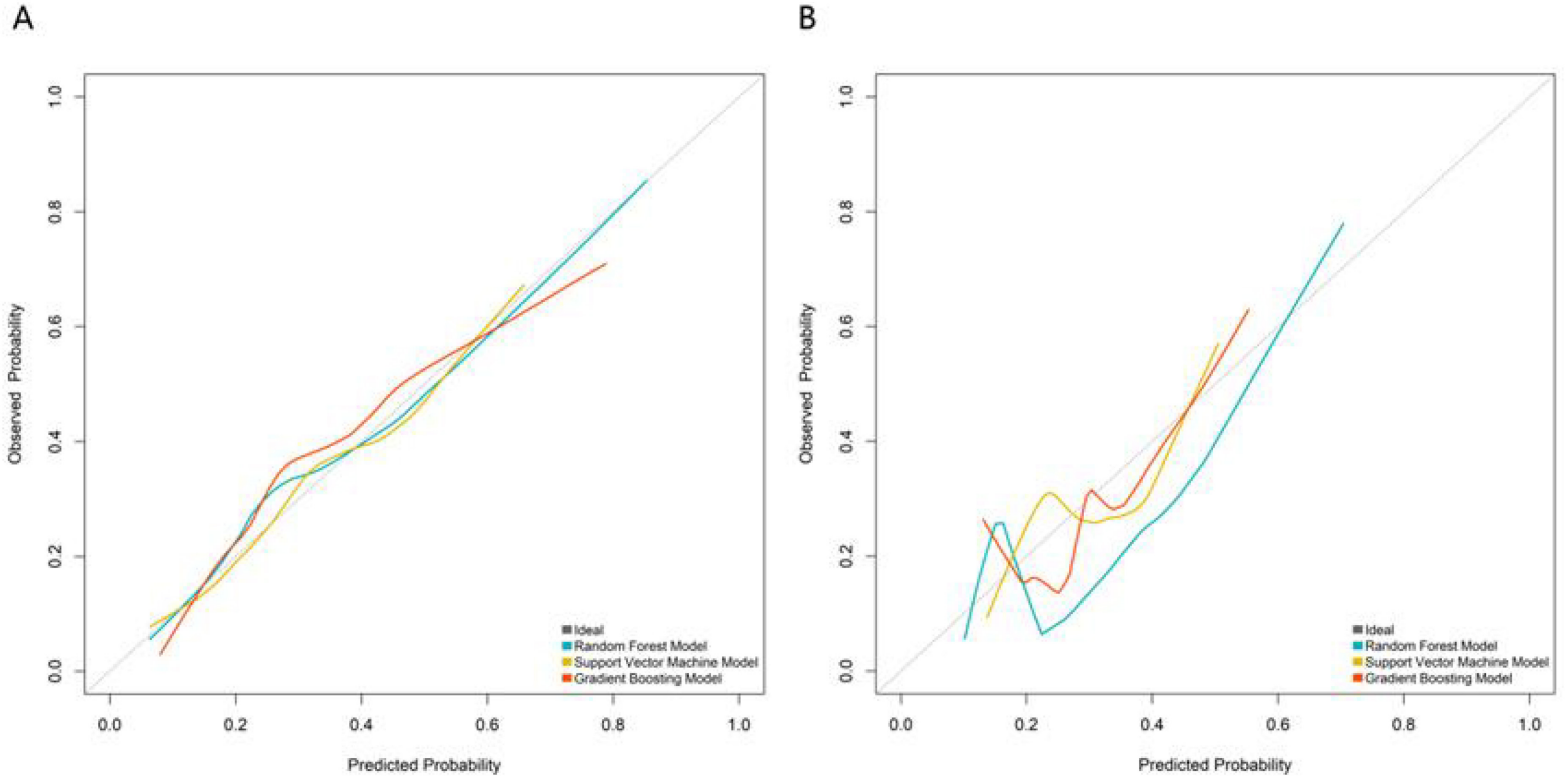
Calibration curves of machine learning models (**A**, the training set; **B**, the validation set).

**FIGURE 4:**
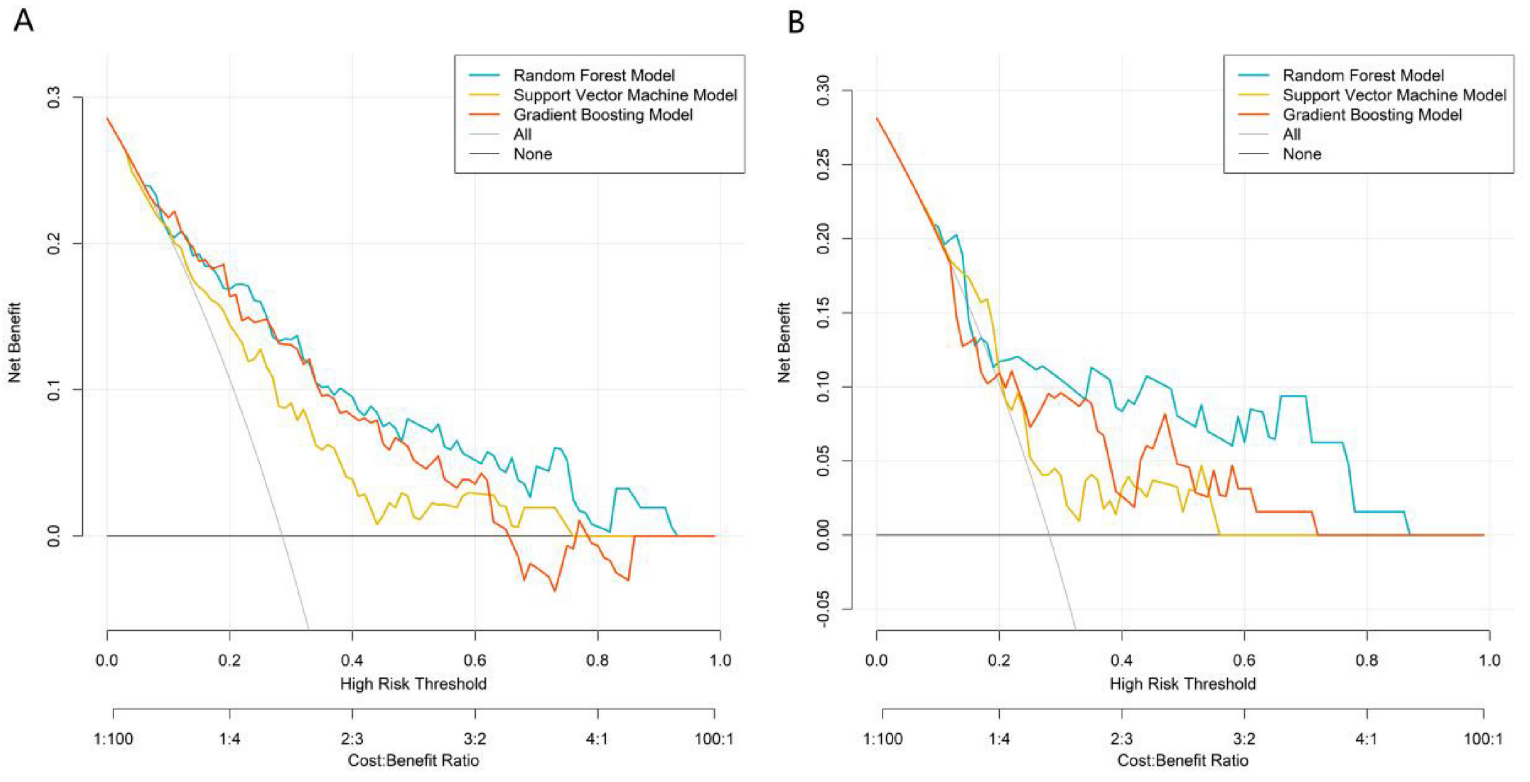
Decision curves of machine learning models (**A**, the training set; **B,** the validation set).

In the training set (Figure 2A), the RF model achieved an AUC of 0.814 (95% CI: 0.738–0.889), the SVM model 0.722 (95% CI: 0.633–0.810), and the GB model 0.805 (95% CI: 0.732–0.879). In the validation set (Figure 2B), the RF model showed an AUC of 0.777 (95% CI: 0.640–0.913), while the SVM and GB models recorded AUCs of 0.665 (95% CI: 0.519–0.812) and 0.682 (95% CI: 0.488–0.835), respectively. These results indicate that the RF model consistently delivered the strongest discriminative performance in both the training and validation phases. The additional performance metrics of the RF model were as follows: in training set, accuracy = 0.789, precision = 0.753, recall = 0.694, F1-score = 0.722, PPV = 0.753, NPV = 0.805; in validation set, accuracy = 0.777, precision = 0.732, recall = 0.679, F1-score = 0.705, PPV = 0.732, NPV = 0.791. These metrics confirm the balanced performance of the RF model in identifying true positive and true negative events. In both the training set (Figure 3A) and validation set (Figure 3B), the calibration curve of the RF model was closer to the ideal calibration dotted line, while the curves of the SVM and GB models deviated more. This indicates that the probability of adverse events during propofol combined with remimazolam tosilate anesthesia predicted by the RF model was more consistent with the actual occurrence probability, with better calibration performance. The results of DCA showed that within a wide range of high-risk thresholds, the net benefit curve of the RF model was consistently higher than the “None” and “All” reference lines, and the curve fluctuation was smaller than that of the SVM and GB models (Figure 4). The RF model performed better in terms of discriminative ability, calibration, and clinical net benefit, and it was the optimal model for predicting adverse events during propofol combined with remimazolam tosilate anesthesia in this study.

### Interpretability evaluation of model prediction results

**FIGURE 5:**
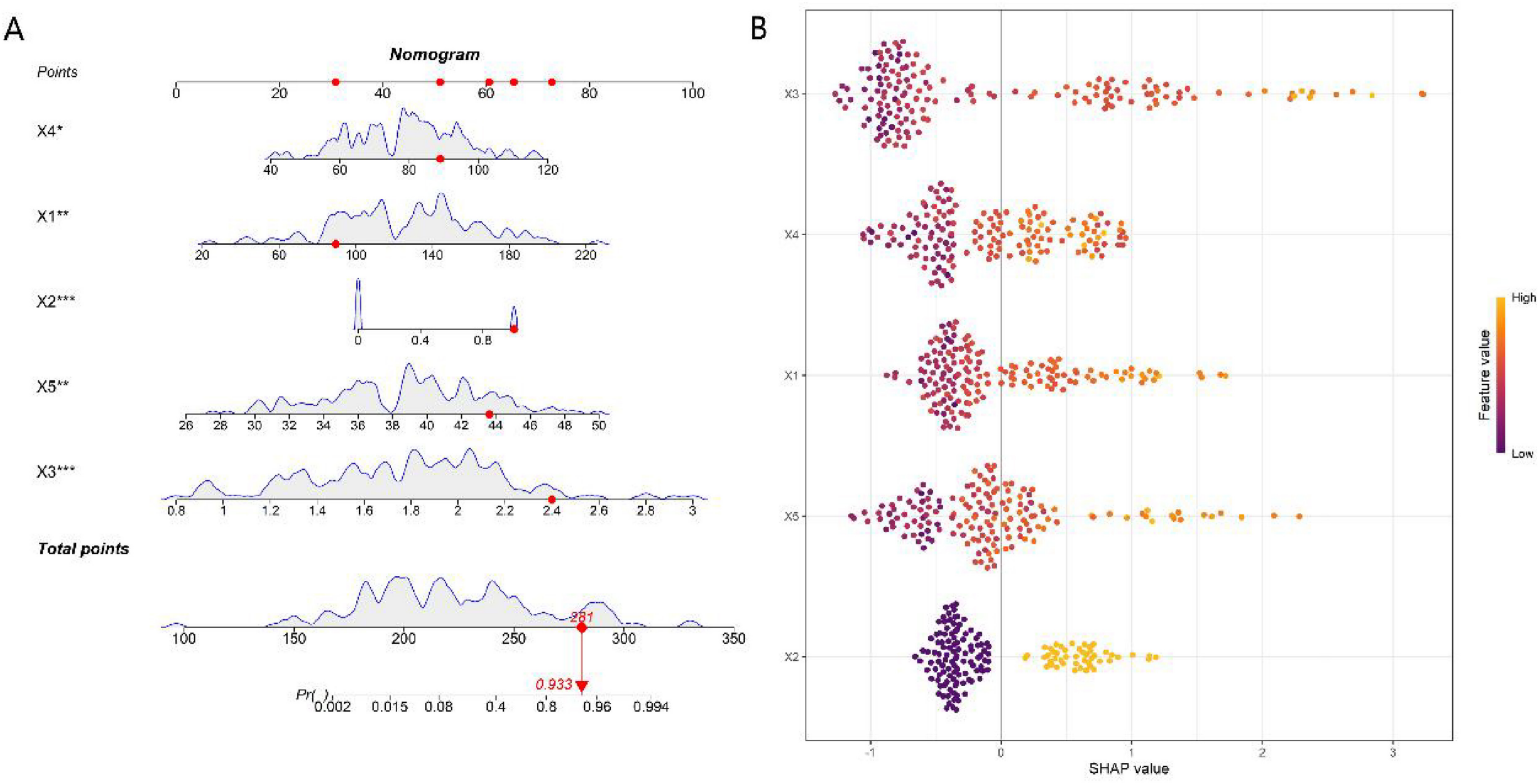
Fancy nomogram **(A)** and SHAP feature importance plot **(B)** (X1, Surgical duration; X2, Intraoperative hypotension incidence; X3, Anesthetic drug dosage ratio; X4, Scr; X5, PaCO_2_).

The nomogram integrates five core features (Figure 5A). Each feature corresponds to a score axis: higher values of the feature correlate with a higher score. By summing the scores of all features to get the total score, clinicians can directly map this total score to the Pf (probability of adverse events) axis at the bottom of the figure.

Shapley value analysis illustrated the impact of each feature on the model’s predictions (Figure 5B). Surgical duration contributed most significantly to the prediction of occurrence of adverse events. A prolonged surgical duration and an elevated anesthetic drug dosage ratio (higher relative proportion of propofol) were identified as key risk factors.

## Discussion

Safety management during general anesthesia is a core aspect of ensuring the prognosis of surgical patients. It is of great significance for achieving individualized anesthesia regimens and enhancing peri-operative safety to accurately predict the risk of adverse events under propofol combined with remimazolam tosilate anesthesia based on objective peri-operative indicators (10). Based on the clinical data of 312 patients, this study integrated intraoperative vital signs, anesthesia recovery indicators, and basic laboratory tests. Through LASSO regression and multivariate Logistic regression analysis, the independent risk factors for adverse events during anesthesia were identified as surgical duration, intraoperative hypotension incidence, anesthetic drug dosage ratio, Scr, and arterial blood PaCO_2_. A prediction model with the random forest model as the optimal one was constructed (AUC = 0.832 in the training set). Among them, surgical duration and anesthetic drug dosage ratio ranked among the top two in the feature importance ranking. This provides important quantitative evidence for anesthesiologists’ preoperative risk assessment (based on preoperatively available indicators such as anesthetic drug dosage ratio) and intraoperative precise regulation (based on dynamically updated intraoperative monitoring indicators).

In this study, surgical duration, which reflects surgical trauma and continuous stress, was identified as the strongest predictor. Its mechanism can be explained by the cascade effect of neuroendocrine and inflammatory responses triggered by surgical trauma. A long-term operation means continuous tissue damage, fluid loss, and exposure to anesthetic drugs, which directly leads to the continuous activation of the hypothalamus-pituitary-adrenal axis, an increase in the levels of stress hormones such as catecholamines and cortisol, and exacerbates the body’s metabolic disorders and oxygen consumption (11). This not only increases the cardiac load, making it prone to myocardial ischemia and arrhythmia, but may also affect the pharmacokinetics of propofol and remimazolam by changing the volume of drug distribution and metabolic rate, resulting in delayed awakening (12). In addition, a prolonged operation time is often accompanied by a larger volume of fluid infusion and body temperature loss. These factors together disrupt the homeostasis of the internal environment, making it more challenging to maintain a stable anesthesia depth and vital signs. The results of this study clearly indicate that optimizing the surgical process and shortening the operation time are key measures to reduce anesthesia risks at the source.

Intraoperative hypotension incidence, a newly included indicator in this study, was a core predictor of adverse events. Intraoperative hypotension can reduce perfusion pressure of vital organs such as the heart, brain, and kidneys, leading to tissue hypoxia and metabolic disorders, which in turn increase the risk of hemodynamic instability and delayed recovery (13). This result emphasizes the importance of active prevention and treatment of intraoperative hypotension, such as optimizing fluid management before anesthesia, adjusting anesthetic dosage in a timely manner, and using vasoactive drugs appropriately, which are critical to improving anesthesia safety.

Anesthetic drug dosage ratio (propofol/remimazolam tosilate) in this study was one of the core predictors of the model; an increase in its value (i.e., an increase in the relative proportion of propofol) was significantly associated with an increased risk of adverse events (14). This result is highly consistent with clinical cognition—propofol has a relatively stronger inhibitory effect on circulation and respiration, while remimazolam tosilate has higher safety, and the dosage ratio of the two directly affects the depth of anesthesia and the body’s tolerance (15). The introduction of this indicator not only enriches the prediction dimension but also provides a quantitative basis for the adjustment of individualized clinical anesthesia protocols, that is, optimizing the dosage ratio of propofol and remimazolam tosilate can reduce the risk of adverse events (16).

Among the laboratory indicators, the predictive value of blood creatinine level and arterial blood PaCO_2_ cannot be ignored (17). An increase in blood creatinine, even if it does not reach the clinical diagnostic criteria for renal insufficiency, may indicate sub-clinical renal function impairment (18). This can affect anesthesia safety in two ways: First, decreased renal function may affect the excretion of the main metabolite of remimazolam (zolam propionic acid). Although its activity is extremely low, whether its accumulation will have other effects requires further study; more importantly, patients with renal insufficiency often have water-electrolyte disorders, anemia, and cardiovascular instability, and their compensatory ability to anesthetic drugs and surgical stress is reduced. An increase in arterial blood PaCO_2_ (hypercapnia) directly reflects insufficient alveolar ventilation (19, 20). Intraoperative hypercapnia can cause cerebral vasodilation, increased intracranial pressure, and may induce arrhythmia. At the same time, respiratory acidosis can change the protein binding rate and ionization degree of drugs, potentially affecting drug efficacy (21). These two indicators link the patient’s baseline physiological reserve with the intraoperative state, emphasizing the necessity of optimizing the patient’s internal environment state before surgery.

In this study, the predictive performance of the random forest model (AUC 0.814) was significantly better than that of the support vector machine and gradient boosting models. Its advantage lies in the high adaptability of its algorithm to the complex characteristics of clinical data. First, the random forest can effectively capture the complex non-linear relationships and interactions between predictive variables and outcomes, such as the possible synergistic effect between surgical duration and MAP fluctuation amplitude. Second, by constructing multiple decision trees through Bootstrap sampling and integrating the results, the model is not sensitive to single outliers or data noise, enhancing its generalization ability and stability. Finally, the feature importance ranking it provides (such as highlighting the leading role of surgical duration) provides clear priority guidance for clinical decision-making, with direct practical guiding significance. The clinical translation path of this model is clear: Anesthesiologists can input the patient’s six indicators into the model in the pre-operative or early intraoperative period to quickly calculate the individualized risk of adverse events. For high-risk patients, targeted measures can be taken in advance, such as optimizing fluid management, preparing vasoactive drugs, presetting more advanced respiratory monitoring (such as end-tidal carbon dioxide partial pressure monitoring), or arranging beds in the post-anesthesia care unit, thus achieving a qualitative change from passive treatment to active early warning.

This study also has several limitations. First, this is a single-center retrospective study. Although it shows good internal validation, the extrapolation performance of the model still needs to be further verified through multi-center, prospective studies. Second, although the included predictive variables are representative, they do not cover all potential influencing factors, such as surgical type details, major comorbidities, chronic medication history, the influence of genetic polymorphisms on drug metabolism, and more detailed cardiac function indicators (such as echocardiogram data). Due to the retrospective nature of the study, some of these data were not fully recorded in electronic medical records, limiting the clinical completeness of the model. Future studies can integrate multi-omics data to further improve the model accuracy. Third, this study mainly focused on the construction and validation of the prediction model and has not yet applied the model to the clinical workflow and evaluated its actual effectiveness in improving patient outcomes. Subsequent studies should focus on developing a clinical decision-support system and conducting stepped-cluster randomized controlled trials to empirically evaluate the real-world effect of model-guided interventions on reducing the incidence of adverse events during anesthesia. Fourth, this study used a composite endpoint (hemodynamic instability, respiratory depression, delayed recovery) to assess anesthesia-related adverse events (ARAEs). Although this can comprehensively reflect anesthesia safety, the pathophysiological mechanisms of each endpoint component are different, which may reduce the model’s specificity for predicting a single complication. In this cohort, hemodynamic instability accounted for 51.1% of the composite endpoint (46 out of 90 adverse events), respiratory depression for 28.9% (26 cases), and delayed recovery for 20.0% (18 cases), with hemodynamic instability being the predominant complication type. The impact intensity of different predictors on each endpoint component may vary (intraoperative hypotension incidence may have a more significant predictive effect on hemodynamic instability, while spontaneous breathing recovery time may have a stronger correlation with respiratory depression), but this study did not conduct stratified analysis for a single complication. Future studies can further develop component-specific predictive models to improve the accuracy of clinical interventions. Fifth, although we employed multiple imputation for missing data, the potential bias from unmeasured confounders cannot be completely excluded. Future studies should consider incorporating real-time waveform data and genetic factors to enhance model accuracy.

In conclusion, based on six core peri-operative indicators, this study successfully constructed and validated a random forest model for efficiently predicting adverse events during propofol combined with remimazolam tosilate anesthesia. The model confirms that surgical duration, hemodynamic stability, and the recovery of respiratory function are the key dimensions for risk prediction. This tool provides strong technical support for achieving precise and proactive anesthesia management and is expected to improve the peri-operative safety level by early identifying high-risk patients and guiding targeted interventions.

## Data Availability

The original contributions presented in the study are included in the article/Supplementary material, further inquiries can be directed to the corresponding author.
