# Supplementary material for: Adverse event prediction in propofol-remimazolam tosilate anesthesia
Source: Front Med (Lausanne). 2026 Jan 15;12:1731100. doi: 10.3389/fmed.2025.1731100 (PMC12852453; doi:10.3389/fmed.2025.1731100)
Supplement: Supplementary file 1 [file Table_1.docx]

**Supplemental Table 1.** Variable assignment table

| Variable | Meaning | Assignment |
| --- | --- | --- |
| X1 | Surgical duration | Continuous variable |
| X2 | MAP fluctuation range | Continuous variable |
| X3 | Minimum duration of SpO₂ | Continuous variable |
| X4 | Time to recovery of spontaneous breathing | Continuous variable |
| X5 | Scr | Continuous variable |
| X6 | PaCO₂ | Continuous variable |
| Y | Whether adverse events occurred | Non-occurrence group=0, occurrence group=1 |
